# Female Sexual Function Before and After Bariatric Surgery: a Cross-Sectional Study and Review of Literature

**DOI:** 10.1007/s11695-015-1721-8

**Published:** 2015-05-20

**Authors:** Michał Robert Janik, Ilona Bielecka, Krzysztof Paśnik, Andrzej Kwiatkowski, Ludmiła Podgórska

**Affiliations:** Department of General, Oncologic, Metabolic and Thoracic Surgery, Military Institute of Medicine, Szaserów 128, 04-141 Warsaw, Poland

**Keywords:** Obesity, FSFI, Sexual dysfunction, Bariatric surgery, Sexual quality of life

## Abstract

**Background:**

The aims of the present study were to compare sexual quality of life and prevalence of female sexual dysfunction (FSD) after surgical weight loss with controls seeking bariatric surgery, and to perform a literature review.

**Methods:**

Female Sexual Function Index (FSFI) and Sexual Quality of Life–Female (SQoL-F) questionnaires were sent within 12–18 months postoperatively via e-mail to 153 women who had undergone weight loss surgery (postoperative group). The control group comprised of 23 women who were asked to complete the questionnaires during their preoperative evaluation (preoperative group). The total FSFI cutoff score for a diagnosis of FSD was ≤ 26.55.

**Results:**

The median (Q1, Q3) FSFI score did not differ significantly between the preoperative (26.9 [24.3, 30.7]) and postoperative groups (26.9 [22.6, 30.0]). There was no difference in the prevalence of FSD between groups. However, median scores in FSFI domains of desire and arousal were significantly higher in the postoperative group. There were no differences in the other FSFI domains. The median SQoL-F was significantly higher in the postoperative group.

**Conclusions:**

The FSFI score did not predict the SQoL-F score. The prevalence of FSD was comparable in the two groups. The higher SQoL-F score in the postoperative group may be the result of an improvement in self-esteem, which in turn leads to greater interest in sex and more intense feelings of desire and arousal.

## Introduction

An increasing incidence of obesity across the USA and Europe has been observed in the last decade [1]. Obesity is accompanied by comorbidities such as type 2 diabetes, hypertension, heart disease, hyperlipidemia, and obstructive sleep apnea [2]. Obesity also reduces the quality of life including sexual quality of life [3]. The negative impact of obesity on general health and socioeconomic issues results in increased financial burden on the entire community [4]. Bariatric surgery is the most effective treatment for obesity and its comorbidities; meta-analysis has confirmed that the efficacy of bariatric procedures is superior to those of current medical therapies [5, 6]. However, little is known about the impact of weight loss after bariatric surgery on sexual quality of life. Female sexual dysfunction (FSD) comprises of impairment of sexual response cycle and pain during or after intercourse [7]; obese women who attempt to qualify for bariatric surgery have been shown to be at higher risk of experiencing FSD [8, 9]. However, the relationship between obesity and disturbances in female sexual function is not well defined. Changes in sexual quality of life and incidence of FSD after bariatric surgery have only been assessed by a few authors, with conflicting results. There is a need to explore this neglected area of bariatric care so that changes in overall quality of life after surgical weight loss can be better understood. The aim of our study was to compare sexual quality of life and the prevalence of FSD before and after the surgical treatment of obesity.

## Materials and Methods

### Study Design and Participants

This cross-sectional study was performed in our Department of Surgery between January and March 2014. The experimental group (postoperative group) comprised of 153 females in the period of 12–18 months after undergoing bariatric surgery, either laparoscopic sleeve gastrectomy (LSG) or laparoscopic Roux-en-Y gastric bypass (LRYGB). The control group (preoperative group) consisted of 23 pre-LSG and pre-LRYGB females. All participants completed a demographic questionnaire and a health history checklist. In the postoperative group, data was collected using an e-mailed questionnaire. The preoperative group was asked to complete the questionnaires during their preoperative evaluation. Informed consent was obtained from all individual participants included in the study.

All participants met the Interdisciplinary European Guidelines on Metabolic and Bariatric Surgery criteria for bariatric surgery [10]. Women who were not sexually active were excluded from the study. Moreover, excluded from the study were patients with a history of medication use, such as antidepressants, psychotropic drugs, beta-blockers, and spironolactone, which can interfere with sexual function.

### Measures

Both groups were asked to complete the Female Sexual Function Index (FSFI) and Sexual Quality of Life–Female (SQoL-F) questionnaires, which are described in detail in Tables 1 and 2.

**Table 1:** Individual FSFI domains: questions and scoring

Domain	Questions	Minimum score	Maximum score	Preoperative group	Postoperative group	*p* value*
Desire	1, 2	1.2	6.0	4.2 (3.0, 4.2)	4.8 (4.5, 5.4)	<0.01
Arousal	3, 4, 5, 6	0	6.0	3.9 (3.6, 4.8)	5.7 (4.8, 6.0)	<0.01
Lubrication	7, 8, 9, 10	0	6.0	5.1 (4.2, 6.0)	4.6 (3.9, 5,7)	0.25
Orgasm	11, 12, 13	0	6.0	4.8 (3.6, 5.2)	4.4 (3.6, 5.6)	0.67
Satisfaction	14, 15, 16	0.8	6.0	4.8 (3.8, 5.6)	4.8 (3.6, 5.2)	0.91
Pain	17, 18, 19	0	6.0	6.0 (3.6, 6.0)	4.0 (3.8, 5.0)	0.21
Total score	2.0	36.0		26.9 (24.3, 30.7)	26.9 (22.6, 30.0)	0.44

*FSFI* Female Sexual Function Index.

**Table 2:** Sexual Quality of Life–Female questionnaire: comparison of item scores between preoperative and postoperative groups

Item	Maximum number of points	Preoperative group	Postoperative group	*p* value*
1. When I think about my sexual life, it is an enjoyable part of my life overall	6	4.7 (3.5, 5.0)	4.0 (4, 6)	0.08
2. When I think about my sexual life, I feel frustrated	6	4.0 (3, 5)	5.0 (5, 6)	<0.01
3. When I think about my sexual life, I feel depressed	6	4.4 (3, 6)	5.0 (4, 6)	0.33
4. When I think about my sexual life, I feel like less of a woman	6	4.3 (2, 6)	4.7 (4, 6)	0.57
5. When I think about my sexual life, I feel good about myself	6	3.3 (2, 5)	4.0 (3, 5)	0.10
6. I have lost confidence in myself as a sexual partner	6	3.4 (2.0, 5.0)	5.1 (4.5, 6.0)	<0.01
7. When I think about my sexual life, I feel anxious	6	3.8 (2.0, 5.0)	5.3 (5.0, 6.0)	<0.01
8. When I think about my sexual life, I feel angry	6	4.0 (3.0, 5.0)	5.2 (5.0, 6.0)	<0.01
9. When I think about my sexual life, I feel close to my partner	6	4.2 (2.0, 6.0)	3.6 (2.0, 5.0)	0.08
10. I worry about the future of my sexual life	6	3.3 (2.0, 5.0)	4.6 (4.0, 6.0)	0.01
11. I have lost pleasure in sexual activity	6	3.7 (4.0, 5.0)	4.6 (4.0, 6.0)	0.16
12. When I think about my sexual life, I am embarrassed	6	3.6 (2.0, 5.0)	5.2 (5.0, 6.0)	<0.01
13. When I think about my sexual life, I feel that I can talk to my partner about sexual matters	6	4.4 (4.0, 6.0)	3.2 (2.0, 4.5)	0.02
14. I try to avoid sexual activity	6	4.2 (3.0, 6.0)	4.6 (4.0, 5.0)	0.58
15. When I think about my sexual life, I feel guilty	6	4.3 (3.0, 6.0)	5.5 (5.0, 6.0)	<0.01
16. When I think about my sexual life, I worry that my partner feels hurt or rejected	6	3.0 (2, 5)	6.0 (5.0, 6.0)	<0.01
17. When I think about my sexual life, I feel like I have lost something	6	4.0 (2.0, 5.0)	6.0 (4.0, 6.0)	<0.01
18. When I think about my sexual life, I am satisfied with the frequency of sexual activity	6	3.3 (2.0, 5.0)	3.1 (2.0, 4.5)	0.73
Total score	108	60.0 (48.8, 73.3)	75.0 (64.4, 82.2)	0.04

Data are presented as median (Q1, Q3)

*p< 0.05 is statistically significant

The FSFI was introduced by Rosen et al. [11]. It is a 19-item self-report questionnaire that assesses the level of functioning in the past 6 months across six domains: (1) sexual desire, (2) sexual arousal, (3) lubrication, (4) orgasm, (5) sexual satisfaction, and (6) sexual pain. Higher scores for each domain and higher total scores indicate better sexual function. The FSFI is widely accepted and used to assess disturbances in female sexual function. Lack of sexual activity, including *no sexual activity* and *did not attempt intercourse*, was defined as a negative answer for most of the 15 items. The total FSFI cutoff score for the diagnosis of FSD in bariatric patients was ≤26.55 [8].

The SQoL-F is an 18-item self-report questionnaire developed by Symonds et al. [12]. It assesses the impact of sexual dysfunction on a woman’s sexual quality of life. Each question is scored on a six-point scale ranging from *completely agree* to *completely disagree*. A higher total score reflects a better sexual quality of life.

Demographic characteristics of age, weight, height, and marital status were collected using an additional questionnaire.

### Statistical Analysis

Statistical analyses were conducted using SAS® software, Version 9.3 (SAS Institute Inc., Cary, NC, USA). Fisher’s exact test was used to determine differences in the prevalence of FSD between groups. The unpaired *t* test and the Mann–Whitney *U* test were used to assess differences in participants’ scores. Pearson’s correlation coefficient was used to assess the relationships of body mass index (BMI) to FSFI total score and of BMI versus SQoL-F total score. Statistical significance was set at *p* < 0.05.

### Literature Review

A review of articles published within the last decade was conducted. Studies were identified by searching the PubMed database for the phrases: *female sexual dysfunction index* and *bariatric surgery*. We included only English-language articles presenting cohort studies that employed the FSFI questionnaire. Data regarding the prevalence of FSD and the total FSFI score were obtained from the studies.

## Results

The survey response rate was 85 % in the preoperative (control) group and 20 % in the postoperative (experimental) group. The groups were comparable in age and smoking status but differed significantly in BMI, comorbidities, hometown population, and marital status. The preoperative group reported comorbidities of hypertension, diabetes, hypothyroidism, hyperlipidemia, sleep apnea, and coronary artery, whereas the postoperative group reported only hypertension. There were four categories regarding marital status: married, divorced, cohabitating, and single. The preoperative group had fewer married women (57 vs. 64 %) and more single women (19 vs. 11 %) than the postoperative group. However, when *married* and *cohabiting* were combined under *partnered* status, the groups were comparable (preoperative 76 % vs. postoperative 78 %). Women in the postoperative group more often lived in small cities (<100,000 inhabitants). In the postoperative group, 64 % underwent LRYGB and 36 % underwent LSG. Patient characteristics are shown in Table 3.

**Table 3:** Sociodemographic characteristics of patients

Characteristic	Preoperative group (*n* = 21/23)	Postoperative group (*n* = 28/31)	*p* value
Age (years)	41 ± 9	44 ± 12	0.14
BMI (kg/m^2^)	46 ± 6	26 ± 3	<0.01
Comorbidities (%)			<0.01
Hypertension	65 %	4 %	
Diabetes	25 %	0 %	
Hyperlipidemia	5 %	0 %	
Hypothyroidism	20 %	0 %	
Obstructive sleep apnea	5 %	0 %	
Coronary artery disease	5 %	0 %	
Marital status (%)			0.02
Partnered	76 %	78 %	
Married	57 %	64 %	
Cohabiting	19 %	14 %	
Divorced	5 %	11 %	
Single	19 %	11 %	
Smoking status (%)			0.05
Never-smoker	56 %	43 %	
Current smoker	18 %	24 %	
Former smoker	26 %	33 %	
Hometown population (%)			<0.01
<100,000	0 %	14 %	
100,000–250,000	19 %	10 %	
250,000–500,000	8 %	10 %	
>500,000	73 %	66 %	

*p* < 0.05 indicates statistical significance

*BMI* body mass index

### Sexual Function and Sexual Quality of Life

**Fig. 1:**
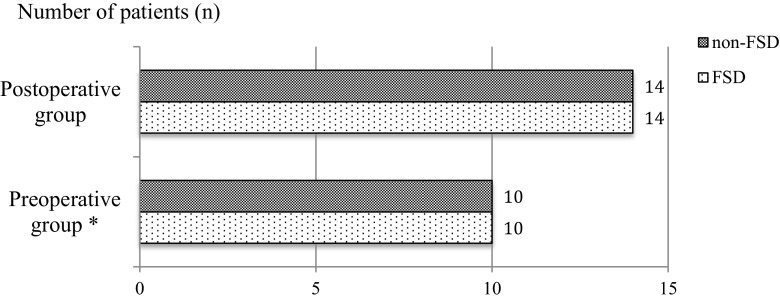
Prevalence of FSD in the preoperative (*n* = 20) and postoperative (*n* = 28) groups. *Incomplete FSFI questionnaire in one case. *FSD* female sexual dysfunction

**Fig. 2:**
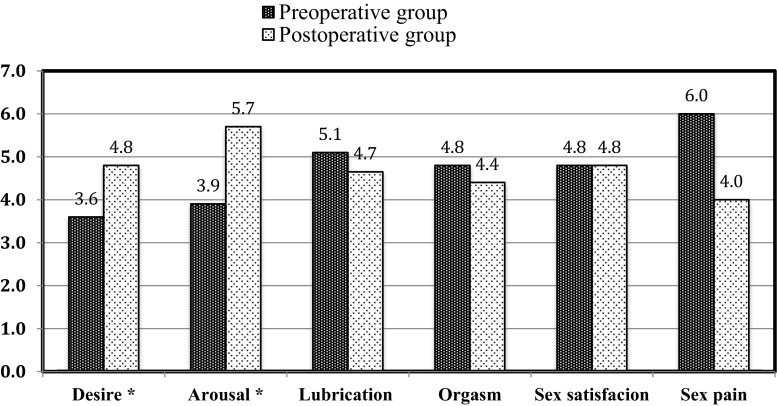
FSFI distribution by domain in preoperative and postoperative groups. *FSFI* Female Sexual Function Index. *p < 0.05

The median (Q1, Q3) total FSFI scores for the preoperative and postoperative groups were 26.9 (24.3, 30.7) and 26.9 (22.6, 30.0), respectively. On the basis of the total FSFI score, FSD was diagnosed in 10 of 20 patients (50 %) in the preoperative group and 14 of 28 (50 %) in the postoperative group (Fig. 1). The lubrication, orgasm, satisfaction, and sex domains did not differ between groups. However, the postoperative group had a significantly higher median in (Q1, Q3) desire (4.8 [4.5, 5.4] vs. 3.6 [3.0, 4.2]) and arousal (5.7 [4.8, 6.0] vs. 3.9 [3.6, 4.8]) scores (Fig. 2 and Table 1). The median (Q1, Q3) total SQoL-F score was significantly higher in the postoperative group (*p* = 0.04, Table 2).

### Associations Between Sexual Function and BMI

There were significant negative correlations between BMI and FSFI total score (*r* = −0.697, *p* < 0.0001) and between BMI and SQoL-F total score (*r* = −0.671, *p* < 0.0001) in the postoperative group. No relationships were observed between BMI and FSFI or SQoL-F score in the preoperative group. Table 4 shows the correlations between BMI and total FSFI and SQoL-F scores for both groups.

**Table 4:** Correlations between BMI and total FSFI and SQoL-F scores by group

		Preoperative group		Postoperative group	
		FSFI total score	SQoL-F total score	FSFI total score	SQoL-F total score
BMI	*r*	–0.362	–0.368	–0.697	–0.671
	*p*	0.15	0.11	<0.0001	<0.0001

*BMI* body mass index, *FSFI* Female Sexual Function Index, *SQoL-F* Sexual Quality of Life–Female

### Literature Review

Six articles met our criteria for inclusion in the literature review. In all, 364 patients underwent bariatric procedures, including RYGB, laparoscopic sleeve gastrectomy, laparoscopic adjustable gastric band, and biliopancreatic diversion. All of these patients were administered the FSFI questionnaire before and after surgery. Results are presented in Table 5.

**Table 5:** Literature review

Author (year)	Surgical procedures	Preoperative				Postoperative					
		*n*	BMI	Total FSFI score	FSD (%)	*n*	BMI	Total FSFI score	FSD (%)	Cutoff for FSD diagnosis	Follow-up (months)
Assimakopoulos et al. (2011)[13]*	BPD-LL, RYGB-LL, SG	59	51.9 ± 9.9	20.7 ± 2.2	–	59	31.8 ± 4.9	25.0 ± 10.3	–	–	12
Bond et al. (2011)[14]^,^	LAGB, RYGB	54	45.1 ± 6.8	24.0 ± 6.3	63	54	–	29.4 ± 4.3	22	≤26.55	6
Lergo et al. (2012)[15]*	RYGB	29	49 ± 7	21.2 ± 9.6	–	9	–	27.1 ± 7.4	–	–	12
Olivera et al. (2012)[16]	LAGB, RYGB, LSG	36	45.76 ± 6.48	17.70 ± 8.38	–	36	31.55	16.91 ± 9.75	–	≤26.0	36
Hernández et al. (2013)[17]*	BPD	80	52.2 ± 8.2	19.9 ± 1.6	–	60	–	30.4 ± 3.5	–	<26.5	12
Sarwer et al. (2014)[18]*	LAGB, RYGB	106	44.5	20.3 ± 10.8	–	103	–	24.8 ± 8.3	–	≤26.0	24

*BPD* biliopancreatic diversion, *BPD-LL* biliopancreatic diversion with long-limb Roux-en-Y reconstruction, *RYGB-LL* Roux-en-Y gastric bypass with long limb, *LAGB* laparoscopic adjustable gastric banding, *LSG* laparoscopic sleeve gastrectomy

*p < 0.05 for difference in mean total FSFI score

## Discussion

This study explored the under-investigated area of female sexual quality of life. In the majority of papers in which FSFI questionnaires were administered, FSD resolved after surgical treatment [13–15, 17, 18]. In contrast, our results do not demonstrate a difference in the prevalence of FSD between the preoperative group and the response-compliant postoperative group. However, a detailed analysis of the FSFI domains revealed significantly higher scores in domains concerning desire and arousal in the postoperative group. The findings are similar to those presented by Olivera et al. [16].

In three recent papers, no significant differences in the prevalence of FSD between healthy controls and obese individuals were observed [19–21], thus challenging the idea that obesity is a risk factor for female sexual dysfunction.

In the present study, however, we observed significantly higher total SQoL-F scores in the postoperative group, indicating a better sexual quality of life in patients who underwent surgery. Unfortunately, because the SQoL-F questionnaire is rarely used in papers on obesity and surgical weight loss, no data is available for comparison. Such contradictory results beg the question, “Why did the postoperative group have a higher mean total score on the SQoL-F questionnaire?”

In our opinion, the different structures of the two measurement methods may explain the results. The SQoL-F consists mainly of questions referring to the emotional aspect of sexual life, such as desire, arousal, self-esteem, anxiety, and satisfaction. As shown in Table 2, women in the postoperative group were less frustrated with their sex life, felt more confident, and were less afraid of hurting or rejecting a partner.

The FSFI questionnaire assesses a wider range of female sexual function, including both emotional (desire, arousal) and physical (lubrication, orgasm, dyspareunia) aspects.

In the present study, women who lost weight after surgery felt more attractive and comfortable with themselves. This improvement in self-esteem and lack of anxiety may have resulted in more interest in sex and including more intense feelings of desire and arousal.

Interestingly, our study revealed significant negative correlations between BMI and total scores for both questionnaires in the postoperative group but not in the preoperative group. Thus, it suggests that the amount of weight loss achieved after surgery affects female sexual quality of life. We postulate that improvements in female sexual quality of life after bariatric surgery may be proportional with the percentage of excess weight loss.

### Limitations

The study had several limitations. First, the number of patients in both groups was small.

Taking into account the fact that we broke down the questionnaires and interpreted them by item, it has to be pointed out that small populations do not guarantee a strong insight. Second, the sensitive nature of the research topic and the restraint of Polish women to talk about sexual activities may have contributed to the low compliance in the postoperative group. However, this low response rate (20 %) corresponds with those in other studies assessing the prevalence of female sexual difficulty and dysfunction, which have ranged from 15 to 33 % with an average response rate of 19 % [22]. Third, because the present study was cross-sectional, a causal relationship between percentage of excess weight loss after bariatric surgery and changes in female sexual quality of life could not be assessed.

It should also be pointed out that body contouring after major weight loss may be a confounding factor, and neither the present study nor the reviewed studies took this into account. We also did not collect data regarding educational status or income. Finally, the cutoff score for the diagnosis of FSD varied from study to study.

## Conclusions

In the present study, the FSFI score did not predict the SQoL-F score. Women who lost weight after bariatric surgery felt more attractive and comfortable with themselves. This improvement in self-esteem and reduction in anxiety may result in more interest in sex and including more intense feelings of desire and arousal.
